# Cancer survival analysis and spatial distribution during 2014–2016 in Shandong Province, China

**DOI:** 10.1038/s41598-023-37252-4

**Published:** 2023-06-26

**Authors:** Fan Jiang, Zhentao Fu, Zilong Lu, Jie Chu, Aiqiang Xu, Xiaolei Guo, Jixiang Ma

**Affiliations:** 1 The Department for Chronic and Non-communicable Disease Control and Prevention, Shandong Center for Disease Control and Prevention, Jinan, China; 2 Institute of Preventive Medicine, Shandong University, Jinan, China

**Keywords:** Cancer epidemiology, Cancer prevention, Cancer

## Abstract

We aimed to analyse cancer survival and its spatial distribution in Shandong Province. A total of 609,861 cancer cases from 2014 to 2016 were included in the analysis. Survival analysis was performed using strs in Stata. Spatial analysis was performed with GeoDa to determine measures of global and local spatial autocorrelation. Hotspot analysis was used to identify spatial clusters of high values (hotspots) and low values (cold spots) through ArcGIS. The 5-year relative survival rates were 37.85% for all cancers combined, 29.29% for males and 48.88% for females. After age standardisation, the survival rates were 34.47% for all cancers, 28.43% for males and 41.56% for females. Cancers with higher survival rates included thyroid (78.80%), breast (69.52%), uterus (64.51%) and bladder (62.54%) cancers. However, cancers with lower survival rates included pancreatic (11.34%), liver (13.19%), lung (18.39%), bone (19.71%), gallbladder (19.78%), oesophagus (24.52%), and stomach (28.85%) cancers and leukaemia (26.30%). Cancer survival rates in urban areas (37.53%) were higher than those in rural areas (32.83%). From the geographic distribution of cancer survival, we observed that the survival rate displayed a downward trend from east to west and from north to south. The hotspot analysis revealed that some counties of Qingdao, Jinan, Zibo, Dongying and Yantai cities were hotspots, whereas almost all counties of Linyi city and some counties of Weifang, Heze, Rizhao, and Dezhou cities were cold spots. In conclusion, the cancer survival rate in Shandong is still lower than that in China overall. The early diagnosis and treatment of lung and digestive tract cancers need to be further strengthened. Nevertheless, our results reflect a critical first step in obtaining and reporting accurate and reliable estimates of survival in Shandong.

## Introduction

Cancer has been the leading cause of death in China for many years. Due to the country’s large population, the global burden of cancer is strongly influenced by the burden of cancer in China^1,2^. According to the GLOBOCAN 2020 report^1^, approximately 23.7% of global cancer cases and 30.2% of global cancer deaths occur in China, with both incidence and mortality rates higher than the global average level. Along with cancer incidence and mortality data, population-based survival estimates provide further insight to assess the effectiveness of cancer prevention and treatment^3^.

International studies have reported survival statistics in developed countries such as the United States, Canada, and Australia for the last 50 years^4–6^. In China, a national-level study reported that the 5-year relative survival rate of cancer was 30.9% during 2003–2005^7^. In 2018, an updated cancer survival study reported that the 5-year relative survival rate increased significantly from 30.9% during 2003–2005 to 40.5% during 2012–2015^8^. These estimates were based on data from only 17 cancer registries covering less than 2% of the Chinese population.

Shandong is the second most populous province in China, with 98.5 million people, accounting for 7.2% of the Chinese population^9^. Three retrospective investigations have been carried out in the province on the total causes of death mainly due to cancer since the 1970s. The results showed that cancer has always been the second leading cause of death in Shandong Province, and the mortality rate is on the rise. Limited by the difficulty of data collection, the abovementioned two cancer survival studies in China covered only 17 cancer registries, including only one registry in Shandong. In addition, some developed provinces in China, such as Shanghai, Zhejiang, and Jiangsu, have carried out population-based cancer survival studies but only reported a single cancer or covered some of the cancer registries^10–12^. To date, the survival rate of cancer has never been reported at the provincial level in Shandong.

The Shandong Center for Disease Control and Prevention is responsible for population-based cancer registration in Shandong Province. In 2011, Shandong Province officially launched cancer surveillance in cancer registration regions. Then, the cancer registries were continuously expanded until 2014, and cancer surveillance covered the whole province. In Shandong, there are 16 cities comprising 139 counties. Therefore, our study aimed to examine cancer survival in Shandong and explore the county-level geographic distribution and spatial clustering of cancer survival.

## Results

### Cancer survival analysis

A total of 609,861 cancer cases diagnosed during 2014–2016 were analysed in Shandong Province, including those of 346,507 males (56.82%) and 263,354 females (43.18%). The age-standardised 5-year survival rates were 34.47% (95% *CI* 34.34–34.60%) for males and females combined, 28.43% (95% *CI* 28.26–28.6%) for males and 41.56% (95% *CI* 41.36–41.76%) for females. The survival rates varied widely according to cancer type, ranging from 78.80% for thyroid cancer to 11.34% for pancreatic cancer in Shandong Province. Cancers with relatively high survival rates (> 60%) included thyroid (78.80%), breast (69.52%), uterus (64.51%) and bladder (62.54%) cancers. However, cancers with low survival rates (< 30%) included pancreatic (11.34%), liver (13.19%), lung (18.39%), bone (19.71%), gallbladder (19.78%), oesophagus (24.52%), and stomach (28.85%) cancers and leukaemia (26.30%), which comprised more than half of all cancer types. The national 5-year survival rates (a total of 659,732 cancer cases diagnosed during 2012–2015 in China^8^) were 40.5% (95% *CI* 40.3–40.7%) for all patients, 33.9% for males (95% *CI* 33.7–34.2%) and 47.8% for females (95% *CI* 47.5–48.1%). In China, cancers with high and low survival rates were similar to those in Shandong. The difference was that survival rates were higher for most types of cancer in China than in Shandong, with the exception of liver, gallbladder, pancreatic, and brain cancers and leukaemia (Table 1).

**Table 1:** Comparions of survival rates between Shandong and China, by cancer site.

Cancer site	ICD-10	Number of patients (%)		Age-standardised relative survival (95%CI)	
		Shandong (2014–2016)	China (2012–2015)	Shandong (2014–2016)	China (2012–2015)
Oral cavity and pharynx	C00-10, C12-14	6939 (1.14%)	7627 (1.16%)	43.32 (41.92–44.72)	50.40 (48.40–52.50)
Nasopharynx	C11	2778 (0.46%)	7966 (1.21%)	40.86 (38.42–43.28)	45.50 (42.60–48.40)
Oesophagus	C15	48,851 (8.01%)	63,506 (9.63%)	24.52 (24.04–24.99)	30.30 (29.60–31.00)
Stomach	C16	78,220 (12.83%)	82,065 (12.44%)	28.85 (28.51–29.19)	35.10 (34.50–35.70)
Colon-rectum	C18-21	49,796 (8.17%)	61,736 (9.36%)	52.39 (51.89–52.89)	56.90 (56.20–57.50)
Liver	C22	59,944 (9.83%)	66,575 (10.09%)	13.19 (12.89–13.50)	12.10 (11.70–12.60)
Gallbladder	C23-24	7497 (1.23%)	10,550 (1.60%)	19.78 (18.76–20.82)	16.40 (15.10–17.60)
Pancreas	C25	11,323 (1.86%)	17,823 (2.70%)	11.34 (10.71–12.00)	7.20 (6.60–7.90)
Larynx	C32	3494 (0.57%)	4029 (0.61%)	47.09 (45.12–49.03)	57.70 (54.80–60.70)
Lung	C33-34	147,270 (24.15%)	122,870 (18.62%)	18.39 (18.17–18.61)	19.70 (19.30–20.10)
Other thoracic organs	C37-38	1465 (0.24%)	1880 (0.28%)	30.92 (28.30–33.58)	36.70 (32.70–40.70)
Bone	C40-41	3356 (0.55%)	3430 (0.52%)	19.71 (18.30–21.17)	26.50 (23.90–29.10)
Melanoma of skin	C43	831 (0.14%)	1305 (0.20%)	43.41 (39.19–47.55)	45.10 (40.10–50.10)
Breast	C50	47,494 (7.79%)	49,176 (7.45%)	69.52 (68.62–70.39)	82.00 (81.00–83.00)
Cervix	C53	12,541 (2.06%)	11,496 (1.74%)	48.94 (47.57–50.29)	59.80 (57.10–62.50)
Uterus	C54-55	10,999 (1.80%)	11,531 (1.75%)	64.51 (62.87–66.09)	72.80 (70.50–75.00)
Ovary	C56	8380 (1.37%)	8576 (1.30%)	36.98 (35.60–38.36)	39.10 (37.20–41.00)
Prostate	C61	7064 (1.16%)	11,690 (1.77%)	52.37 (50.34–54.36)	66.40 (63.70–69.00)
Testis	C62	400 (0.07%)	579 (0.09%)	57.16 (48.47–64.93)	55.20 (42.50–67.80)
Kidney	C64-66, C68	9777 (1.60%)	15,671 (2.38%)	58.18 (56.96–59.38)	69.80 (68.50–71.10)
Bladder	C67	11,346 (1.86%)	16,727 (2.54%)	62.54 (61.51–63.56)	72.90 (71.60–74.10)
Brain	C70-72	15,126 (2.48%)	10,391 (1.58%)	36.76 (35.84–37.68)	26.70 (25.10–28.20)
Thyroid	C73	22,737 (3.73%)	18,470 (2.80%)	78.80 (76.94–80.52)	84.30 (81.80–86.80)
Lymphoma	C81-85, C88, C90, C96	10,516 (1.72%)	16,903 (2.56%)	32.21 (31.20–33.22)	37.20 (36.00–38.40)
Leukaemia	C91-95	11,560 (1.90%)	13,190 (2.00%)	26.30 (25.28–27.32)	25.40 (24.10–26.80)
All others	NA	20,157 (3.31%)	23,970 (3.63%)	45.64 (44.83–46.46)	53.30 (52.20–54.40)
All sites	C00-97, D32-33, D42-43, D45-47	609,861 (100%)	659,732 (100%)	34.47 (34.34–34.60)	40.50 (40.30–40.70)

Including data for patients. ICD-10 = International Classification of Diseases, tenth revision. NA = not applicable.

The national data including number of patients and survival rates come from 17 cancer registries in China. Patients diagnosed during 2012 to 2015.

**Figure 1:**
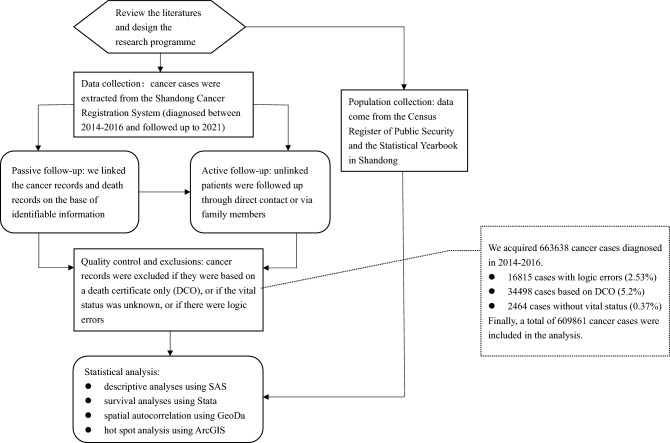
Flow diagram of the study design and methodology.

**Figure 2:**
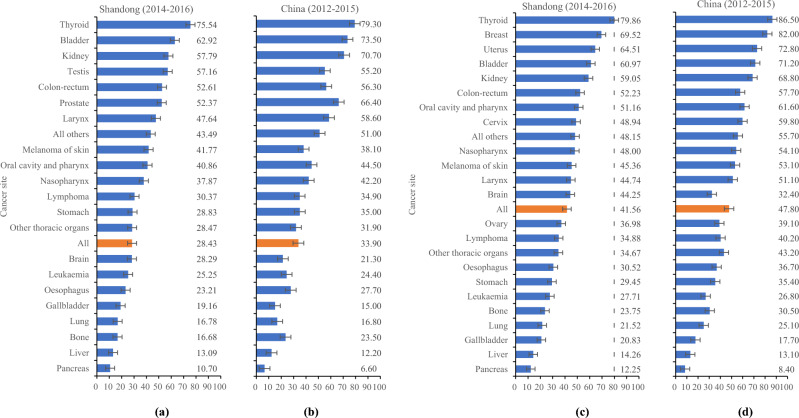
Age-standardised 5-year relative survival rates in Shandong and China by sex; error bars show 95% CIs. (**a**) Survival rates in Shandong during 2014–2016 for males; (**b**) survival rates in China during 2012–2015 for males; (**c**) survival rates in Shandong during 2014–2016 for females; (**d**) survival rates in China during 2012–2015 for females.

We further examined the cancer survival profiles for males and females. The age-standardised 5-year relative survival rates are presented for 22 cancers in males and 24 in females (Fig. 1). In Shandong, female patients had a higher survival rate than male patients for almost all cancer types, except for bladder, colorectal, and larynx cancers. For males, the five types of cancer with better prognoses were thyroid cancer (77.54%), bladder cancer (62.92%), kidney cancer (57.79%), testicular cancer (57.16%) and colorectal cancer (52.61%), and the five types of cancer with poor prognoses were pancreatic cancer (10.70%), liver cancer (13.09%), bone cancer (16.68%), lung cancer (16.78%), and gallbladder cancer (19.16%). For females, the five types of cancer with better prognoses were thyroid cancer (79.86%), breast cancer (69.52%), uterine cancer (64.51%), bladder cancer (60.97%) and kidney cancer (59.05%), and the five types of cancer with poor prognoses were pancreatic cancer (12.25%), liver cancer (14.26%), gallbladder cancer (20.83%), lung cancer (21.52%) and bone cancer (23.75%). In China, female patients also had better survival rates than male patients for almost all cancer types, except for cancers of the kidney, bladder, and larynx. For both sexes, the distribution of survival rates among different cancer types was similar to that in Shandong (Fig. 2).

In Shandong, survival was lower in rural areas than in urban areas, both for all cancers combined and for major individual cancers (Table 2). The age-standardised 5-year survival rates were 37.54% in urban areas and 32.83% in rural areas. This survival gap between urban and rural areas was even more obvious in China, with estimates of 46.7% in urban areas and 33.6% in rural areas. However, the survival rate of oesophageal cancer in rural areas of Shandong (25.37%) was slightly higher than that in urban areas (23.41%), while in China, the difference between rural and urban areas was more marked (33.20% vs. 18.10%). In addition, the survival rate of cervical cancer in rural areas of China (62.90%) was higher than that in urban areas (54.70%).

**Table 2:** Survival rates for all cancers combined and major cancers between urban and rural areas, in Shandong and China.

Cancer sites	Survival in Shandong (2014–2016)		Survival in China (2012–2015)	
	Urban areas (95%CI)	Rural areas (95%CI)	Urban areas (95%CI)	Rural areas (95%CI)
All cancers	37.54 (37.33–37.75)	32.83 (32.66–32.99)	46.70 (46.50–47.00)	33.60 (33.30–33.90)
Lung	20.45 (20.08–20.83)	17.40 (17.13–17.68)	23.80 (23.20–24.30)	15.40 (14.90–15.90)
Stomach	29.94 (29.37–30.51)	28.52 (28.09–28.95)	36.90 (35.90–37.90)	34.40 (33.70–35.10)
Liver	14.28 (13.77–14.80)	12.78 (12.40–13.17)	14.00 (13.30–14.70)	11.20 (10.60–11.80)
Oesophagus	23.41 (22.59–24.24)	25.37 (24.79–25.95)	18.10 (16.70–19.50)	33.20 (32.40–34.00)
Colon-rectum	54.33 (53.56–55.09)	51.30 (50.63–51.96)	59.30 (58.40–60.10)	52.60 (51.40–53.80)
Breast	73.20 (71.86–74.48)	66.86 (65.68–68.02)	84.90 (83.80–86.00)	72.90 (70.50–75.40)
Thyroid	82.77 (79.82–85.34)	75.47 (73.03–77.73)	86.20 (83.30–89.00)	79.00 (73.90–84.10)
Bladder	64.90 (63.27–66.48)	61.18 (59.84–62.49)	76.00 (74.60–77.40)	65.50 (63.00–68.00)
Uterus	68.82 (66.21–71.27)	61.53 (59.45–63.54)	77.50 (74.90–80.00)	60.10 (56.10–64.00)
Cervix	51.02 (48.75–53.25)	47.86 (46.14–49.55)	54.70 (50.90–58.60)	62.90 (59.20–66.60)

**Figure 3:**
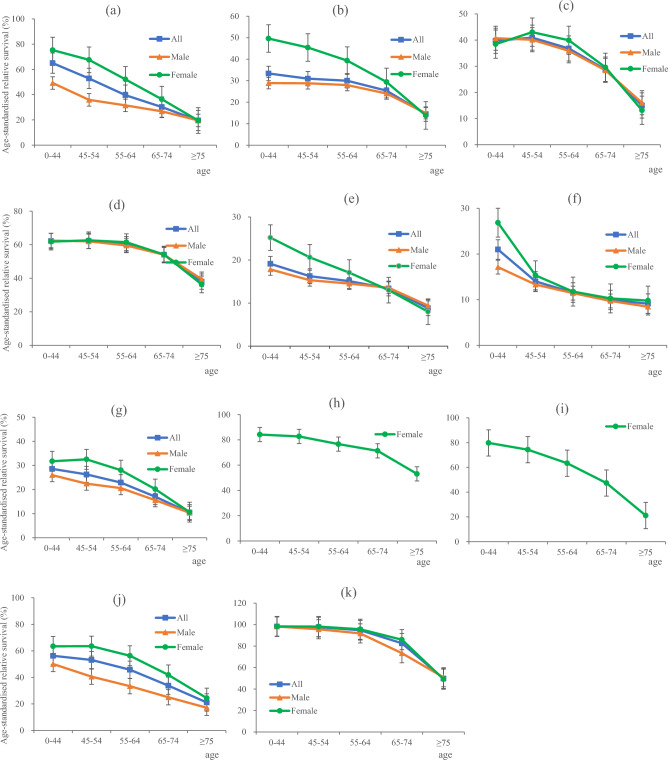
Trends in age-standardised 5-year relative survival rates for all cancers combined and the most prevalent cancers; error bars show 95% CIs. (**a**) Survival trends for all people, males and females for all cancers combined; (**b**)–(**k**) Survival trends for all people, males and females for oesophageal cancer, stomach cancer, colorectal cancer, liver cancer, pancreatic cancer, lung cancer, breast cancer, cervical cancer, brain cancer and thyroid cancer.

The relative survival rate was generally lower for older patients than for younger patients. For all cancers combined, we observed a downward trend in relative survival with increasing age. The 5-year relative survival rate for patients younger than 45 years was 65.01%, whereas for patients aged 75 years and older, the 5-year survival rate was only 19.51%, with an absolute difference of 45.5% between the two groups. Moreover, we also observed similar downward trends in relative survival rates for the ten most prevalent cancers. By cancer type, we observed the largest 5-year survival difference (58.67%) for cervical cancer, with survival rates of 79.78% in the youngest and 21.11% in the oldest age groups (Fig. 3).

### Spatial distribution

**Figure 4:**
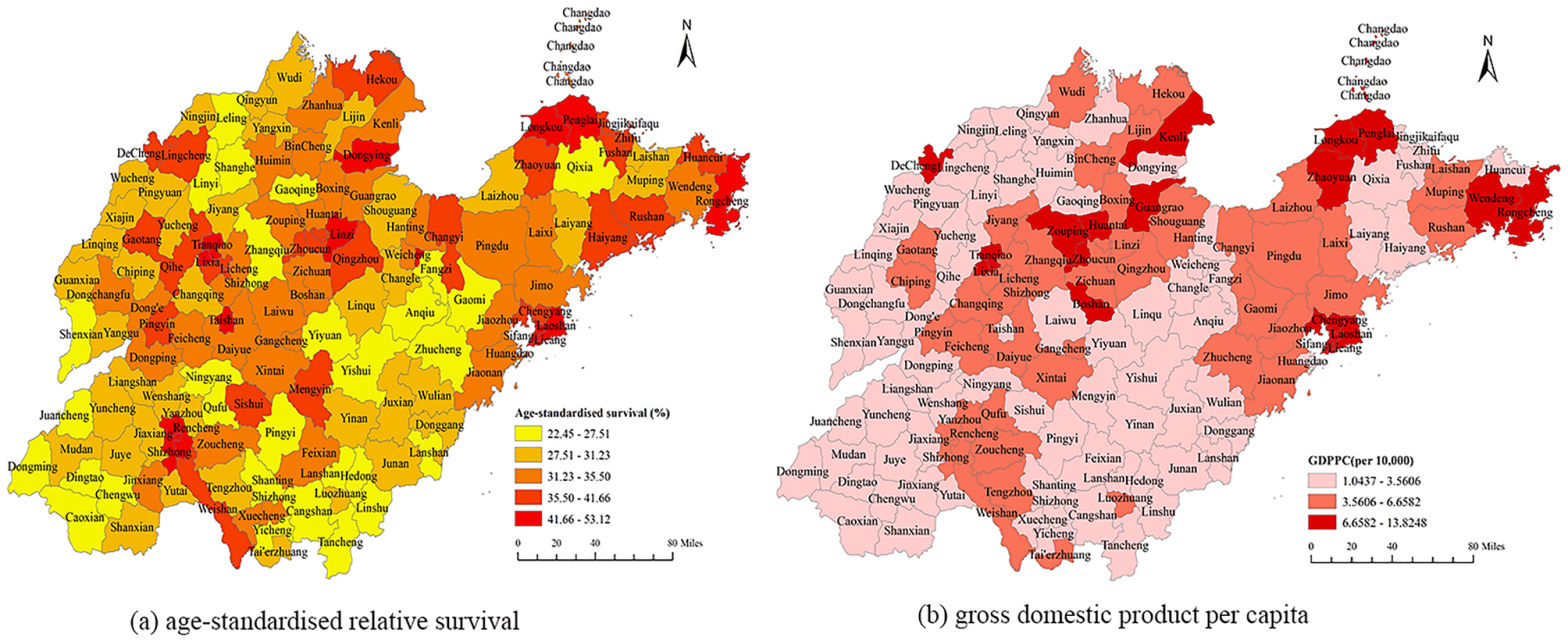
Geographic distribution of cancer survival and economic level in Shandong Province for the years 2014 to 2016. (**a**) Age-standardised relative survival; (**b**) Gross domestic product per capita. Note: the color scale represent the value of survival rate and GDP per capita, the darker the color, the higher the value. Software version number: ArcGIS enterprise v10.8, URL link: https://www.esri.com/zh-cn/arcgis/products/arcgis-enterprise/overview.

The geographic distribution of cancer survival in Shandong Province (Fig. 4a) showed that cancer survival varied widely in different counties. The age-standardised 5-year relative survival rate at the county level ranged from 22.45 to 53.12%. In general, cancer survival showed a downward trend from east to west and from north to south. Regions with higher cancer survival rates were mainly concentrated in most counties of Weihai and some counties of Qingdao and Yantai, which are located in eastern Shandong; some counties of Jinan and Zibo and one or two counties of Weifang and Taian, which are located in central Shandong; some counties of Dongying, which is located in northern Shandong; and one or two counties of Jining and Dezhou, which are located in southwestern and northwestern Shandong, respectively. However, lower cancer survival rates were concentrated in most counties of Linyi and Rizhao and some counties of Zaozhuang, which are located in southern Shandong; most counties of Weifang and one or two counties of Jinan, which are located in central Shandong; and most counties of Heze and some counties of Dezhou and Liaocheng, which are located in western Shandong.

The county-level distribution of GDPPC in Shandong is displayed in Fig. 4b and is classified into high, middle and low levels, with the red colour changing from dark to light. The map shows that the distributions of GDPPC and cancer survival are mostly consistent. Regions with higher economic levels are mainly distributed in most counties of Qingdao, Yantai and Weihai and some counties of Dongying, Jinan, Zibo and Taian; however, regions with lower economic levels are located in most counties of Dezhou, Liaocheng, Heze, Rizhao and Linyi and some counties of Weifang and Jining. In general, the economic levels in Shandong are high in the east and low in the west.

### Hotspot analysis

Moran’s I provided us with some insights regarding the overall and local spatial autocorrelation in cancer survival. As a result, the global and local Moran’s I values were 0.362 and 0.293, respectively, with both p values less than 0.01, showing that there was spatial autocorrelation of cancer survival rates overall and at the province level. Hence, we further conducted hotspot analysis based on the Getis-Ord Gi* statistic.

**Figure 5:**
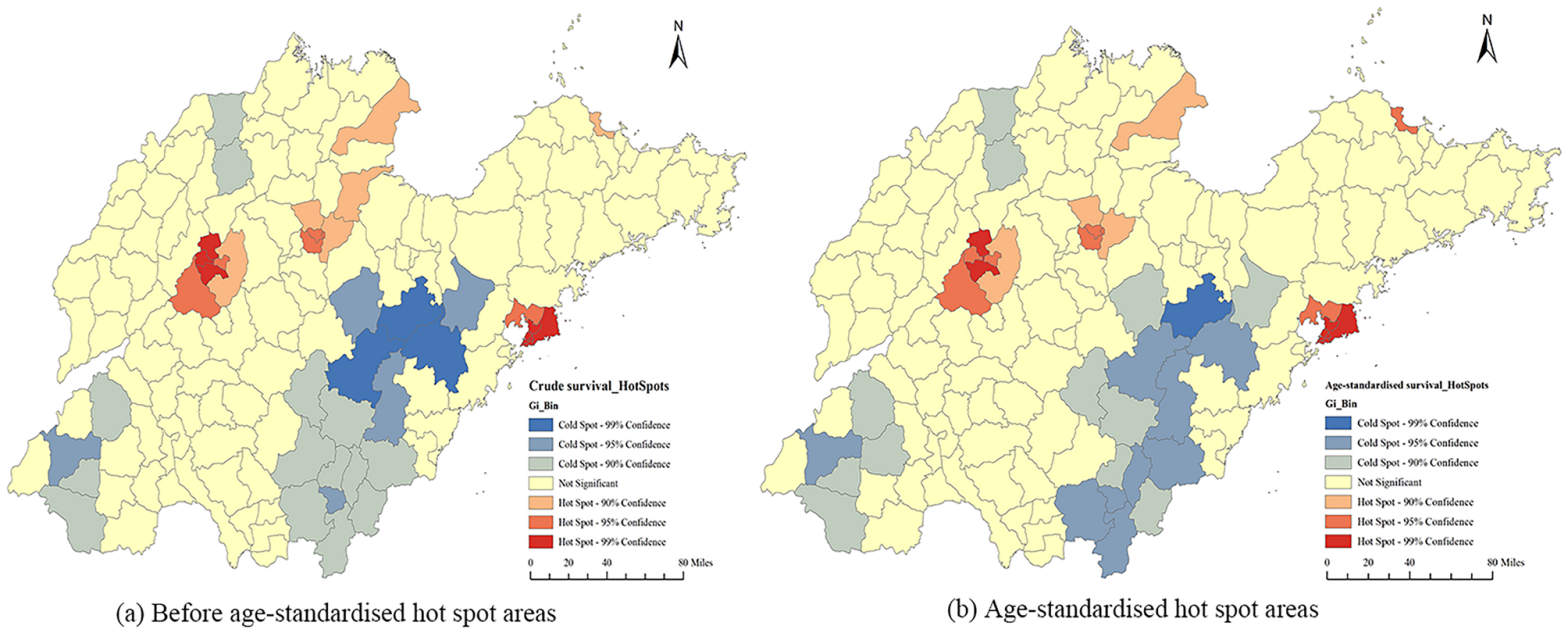
Hot spot areas of cancer survival at the county level in Shandong for the years 2014 to 2016. (**a**) Before age-standardised hot spot areas; (**b**) Age-standardised hot spot areas. Software version number: ArcGIS enterprise v10.8, URL link: https://www.esri.com/zh-cn/arcgis/products/arcgis-enterprise/overview.

As shown in Fig. 5a,b, some counties of Qingdao city, including Shinan, Shibei, Sifang, Laoshan, Licang and Chengyang; some counties of Jinan city, including Shizhong, Tianqiao, Huaiyin, Lixia, Changqing and Licheng; some counties of Zibo city, including Zhangdian, Gaoxinqu, Huantai and Linzi; Kenli and Guangrao of Dongying city; and Jingjikaifaqu of Yantai city were identified as hotspots (clustering areas of high cancer survival rates). After age standardisation, the hotspots were almost the same for the above counties except for Guangrao, which was no longer a hotspot.

In contrast, almost all counties of Linyi city (except for Pingyi); some counties of Weifang city, including Anqiu, Zhucheng, Linqu and Gaomi; some counties of Heze city, including Mudan, Dingtao, Caoxian and Yuncheng; Juxian of Rizhao city; Leling of Dezhou city; and Shanghe of Jinan city were cold spots (clustering areas of low cancer survival rates). After age standardisation, the cold spots were almost the same as before. Juye of Heze city was a new cold spot, while Feixian of Linyi city was no longer a cold spot.

## Discussion

Since 2015, China has implemented a series of plans and policies to control cancer in view of its current severe situation. Cancer survival is considered a key indicator for evaluating the level of cancer prognosis and the effectiveness of health care. This study is the first analysis to explore the overall profile of cancer survival and, moreover, to explore the geographic distribution and spatial clustering of cancer survival at the county level in Shandong Province, the second most populous province in China.

Here, we reported that the age-standardised 5-year relative survival rate for all cancers combined diagnosed during 2014–2016 in Shandong was 34.5%, much lower than the age-standardised survival rate of 40.5% during 2012–2015 in China^8^. One reason for the difference in the survival rate for all cancers combined between Shandong and China relates to the differences in cancer profiles. In Shandong, age-standardised 5-year relative survival rates for the most common cancers (lung, stomach, liver, colorectal and oesophageal), which account for the highest proportion in all cancers combined, were almost below 30%, except colorectal cancer (52.4%). However, in China, 5-year survival rates for stomach, oesophageal and colorectal cancers were more than 30%, and, except for liver cancer (12.1% in China vs. 13.2% in Shandong), the survival rates of other common cancers (19.7% vs. 18.4% for lung cancer; 35.1% vs. 28.9% for stomach cancer; 56.9% vs. 52.4% for colorectal cancer; 30.3% vs. 24.5% for oesophageal cancer) were higher than those in Shandong. Another reason might be that the survival rate of 40.5% reported in China was based on survival data from 17 registries of areas with higher economic levels than the national average level; as a result, the real survival rate of the whole country may be less than 40.5%. These reasons may partly explain the lower survival rates for all cancers combined in Shandong Province.

Regarding cancer type, relative survival rates for most individual cancers were also substantially lower in Shandong than in China, with the exceptions of liver (13.2%), pancreatic (11.3%), gallbladder (19.8%), testicular (57.2%), and brain (36.8%) cancers and leukaemia (26.3%), for which survival rates were higher than those in China (12.1% for liver, 7.2% for pancreatic, 16.4% for gallbladder, 55.2% for testicular, and 26.7% for brain cancers and 25.4% for leukaemia). Hence, these results suggest that our province needs to further strengthen the early diagnosis and treatment of cancers, especially for common cancers such as lung, oesophageal, stomach, liver and colorectal cancers. We also observed a survival disparity between male and female patients for all cancers combined, with female patients generally having better survival rates than male patients. Such gaps should be further investigated using data on clinical factors that might explain these differences.

Compared with other developed counties, the 5-year survival rates of our province and even our country were still lower for all cancers combined and for most individual cancers, with the exception of lung cancer, for which the survival rate was similar. With the same method (Ederer II), age-standardised 5-year relative survival rates were 67% for the USA in 2007–2013 and 68% for Australia in 2009–2013^13,14^. For breast cancer, the survival rates in the USA and Australia exceeded 90% in 2007–2013, while the estimates for China and Shandong were 82.0% in 2012–2015 and 69.52% in 2014–2016, respectively. For thyroid cancer, the survival rates in Shandong and China were 78.8% and 84.3%, respectively, but in the USA and Australia, they were as high as 90% in a similar period. For prostate cancer, the survival rates were 98.2% in the USA and 94.5% in Australia, compared with a low rate of 66.4% in China and an even lower rate of 52.37% in Shandong. It is likely that at least part of these differences are due to differences in screening practices, particularly mammography screening for breast cancer^15^ and prostate-specific antigen testing for prostate cancer, which are more widely used in Western countries than in China. Country-specific differences in management, including initial treatment, surveillance and support networks, are other likely explanations of these survival disparities.

We found regional differences in survival for all common cancers between urban and rural areas, as in a national study^7,8^. The causes for this marked rural‒urban disparity in cancer survival are not fully understood, but the poor level of treatment and limited medical conditions in rural areas may be important contributors. In addition, we observed that survival for oesophageal cancer was higher in rural areas than in urban areas. In response to the national call for early diagnosis and treatment of oesophageal cancer, 14 counties and districts in Shandong Province have carried out early screening for oesophageal cancer. Although this practice is also carried out in urban areas, it started relatively late and covers a smaller population than that in rural areas. Therefore, early diagnosis and treatment of cancer have been more effective in rural areas where the survival rate for oesophageal cancer has substantially improved.

The difference in survival by age is an important public health issue that needs further attention. In our analysis, we observed that the survival rates of most cancers decreased with age, indicating a considerable survival disadvantage for the elderly population (≥ 65 years), similar to that observed in China and many other countries^8,16–18^. This is because elderly individuals are more likely to be diagnosed in the middle or late stages, and they are not as willing to be treated as are young people. Furthermore, the elderly population has more comorbidities, such as heart and cerebrovascular comorbidities and other factors, resulting in the survival of elderly patients being less optimistic than that of young patients^19,20^.

In addition, we conducted a geographic analysis of cancer survival at the county level in Shandong Province and detected survival disparities in different areas. Areas with higher cancer survival rates were found in predominantly eastern and central cities with better economic levels, for example, most counties of Qingdao, Yantai and Weihai and some counties of Jinan, Zibo, Taian and Weifang, whereas areas with lower cancer survival rates were found in mostly southern and western cities with poorer economic levels, including most counties of Linyi, Zaozhuang, Rizhao and Heze and some counties of Dezhou and Liaocheng.

Hotspot analysis based on the Getis-Ord Gi* statistic showed that the hotspots were located in some counties of Qingdao, Jinan, Zibo, Dongying and Yantai cities, whereas almost all counties of Linyi city and some counties of Weifang, Heze, Rizhao, and Dezhou cities were identified as cold spots. International studies have reported poorer cancer survival rates among patients of lower social classes or living in deprived areas and better survival among patients of higher social classes or living in affluent areas^21–23^. Local governments in affluent areas with a high GDP are more likely to allocate a larger proportion of total expenditures on health care, so patients may have easier access to health resources and better medical services^24^. Patients living in areas with low socioeconomic levels usually cannot afford medical services. They may have their disease diagnosed in a later stage and have suboptimal or even no treatment. These factors could lead to a higher mortality risk within the short term after diagnosis. Therefore, in Shandong, more developed cities with higher socioeconomic levels, such as Qingdao, Jinan, Zibo, Dongying and Yantai, have better cancer survival rates than less developed cities with lower socioeconomic levels, such as Heze, Rizhao, Dezhou and Linyi.

Our study was a provincial-level cancer survival study that covered the whole population, which can provide more reliable and robust survival data. In general, the 5-year cancer survival rate in Shandong was lower than the national average level. Survival rates varied by cancer profile. Early diagnosis and treatment of cancers with poor survival rates, for example, lung, oesophageal, gastric, liver and colorectal cancer, need to be strengthened. Cancer survival rates in urban and eastern areas were higher than those in rural and western areas. The difference in economic level leads to the lack of adequate medical care in rural and less affluent counties in the west, so the government should increase investment in medical care in rural and western areas to narrow this gap.

Some limitations of this study should be noted. Population-based cancer registries currently do not have access to a high proportion of pathologic typing, clinical staging or treatment-related information, so we could not study this more in depth. In the future, we will further improve the collection of information on pathologic staging and treatment-related data to better inform cancer prevention and control and improve the prognosis of cancer patients. In spite of this, the survival estimates reported in this study reflect a critical first step in obtaining and reporting accurate and reliable estimates of survival in Shandong. These results will serve as a baseline for future comparisons and assessments to better understand the overall effectiveness of cancer health care in Shandong.

## Methods

### Study population and data collection

Cancer registration data were extracted from the Shandong Cancer Registration System (SCRS). All cancer patients were diagnosed between January 1, 2014, and December 31, 2016, and followed up until December 31, 2021. We obtained the vital status information of cancer patients using a mix of active and passive methods. First, we linked cancer records and death records on the basis of identifiable information. Unlinked patients were further followed up through direct contact or via family members. All cancer cases were classified according to the International Classification of Diseases for Oncology, 3rd ion (ICD-O-3) and the International Statistical Classification of Diseases and Related Health Problems, 10th Revision (ICD-10). The final dataset included variables describing demographic characteristics, date of diagnosis, anatomical site, morphology, behaviour code, vital status, and last date of contact. The population data and gross domestic product per capita (GDDPC) data came from the Census Register of Public Security and the Statistical Yearbook in Shandong.

### Quality control and exclusions

The quality and completeness of the cancer registration data were assessed with IARC-crgTools^25^ to identify errors, inconsistencies and unusual combinations of cancer site, morphology, sex and age at diagnosis. Questionable records were sent back to the cancer registry for verification and correction. Cancer records were excluded if they were based on a death certificate only (DCO), if the vital status was unknown, or if there were logic errors. Therefore, among the 663,638 cancer cases diagnosed in 2014–2016, there were 16,815 cases with logic errors (2.53%), 34,498 cases based on a DCO (5.2%) and 2464 cases without vital status information (0.37%). Finally, a total of 609,861 cancer cases were included in the analysis.

### Statistical analysis

We used relative survival (RS) as the main survival indicator, which was calculated as the ratio of the observed survival of the cancer patients and the expected survival of the general population. Survival was estimated with the classic cohort approach, in that all patients had at least 5 years of potential follow-up. Observed survival was calculated by the life table method, and expected survival was estimated by the Ederer II model^26^. Abridged life tables were stratified by age, sex, and calendar year, smoothed to complete life tables and extended to the age of 99 years using the Elandt-Johnson method^27^. We estimated survival separately for 22 cancers in males and 24 in females. Cancers of the colon and rectum were analysed together, as were all oral and pharyngeal cancers. The remaining cancers were analysed as a single group. We classified areas as urban or rural based on standards set by the National Bureau of Statistics in China^28^. According to the International Cancer Survival Standards, cancer patients were divided into five major age groups (0–44, 45–54, 55–64, 65–74 and 75–99)^29^. We used standard weights to calculate age-standardised relative survival: 7% (0–44 years), 12% (45–54 years), 23% (55–64 years), 29% (65–74 years), and 29% (75–99 years). The same age weights were used for male and female patients, for all cancers combined, and for each area and individual cancer, enabling direct comparison of age-standardised relative survival between patient groups. We calculated standard errors by the Greenwood formula, assuming a normal distribution, and derived 95% *CIs* accordingly^30^. Descriptive analyses were conducted using SAS (version 9.4), while survival analyses were performed using strs^31^ in Stata (version 16.0) software.

Spatial data analysis was performed through GeoDa software (version 1.18.0.0)^32^ to determine measures of global and local spatial autocorrelation. Spatial correlation is the correlation between observations of a single variable solely attributable to their proximity in space. Spatial autocorrelation measurements and tests can be differentiated by the range or scale of analysis, as distinguished from global and local measures^32,33^. Both measurements indicate the degree of spatial association of the dataset. To evaluate the existence of spatial autocorrelation, we used the queen contiguity matrix, which allows for the measurement of a non-random association between the value of a variable observed in a given geographical unit and the values of variables observed in neighbouring units^34,35^. Moran’s I calculates spatial autocorrelation as a covariance from the product of the deviations from the mean^33^. This index calculates the spatial association present in the dataset within all locations under consideration. Moran’s I varies from − 1 to 1, where − 1 means perfect dispersion, 0 represents random behaviour and 1 means perfect association. For all given sets of locations and an associated attribute, it evaluates whether the pattern expressed is clustered, dispersed, or random^36^. The local version of Moran’s index and the Getis-Ord Gi* statistic^37^ were used to identify spatial clusters of high values (hotspots) and low values (cold spots). A high value of the Getis-Ord statistic represents a group of high index values (hotspots), while a low value represents a low value of the index group. Hotspot analysis was performed through ArcGIS software (version 10.8). All reported probabilities (*p* values) were two-sided, and values less than 0.05 were considered statistically significant. Figure 1 shows a flow diagram of the study design and methodology.

### Ethical approval

All methods in our study were carried out in accordance with relevant guidelines and regulations. The entire research protocol was approved by the Ethics Committee of Preventive Medicine in the Shandong Center for Disease Control and Prevention in 2013, with approval no. 2013020. All subjects and/or their legal guardians provided verbal informed consent.

## Data Availability

We acquired the data through official surveillance of the Shandong Death Registration System (SDRS). All data generated and analysed during the course of this study were available from the corresponding author upon request.
